# Psychometric properties of the Greek versions of the Pandemic-Related Pregnancy Stress Scale and the Pandemic-Related Postpartum Stress Scale and associated risk factors during the second year of the COVID-19 pandemic

**DOI:** 10.1192/bjo.2022.635

**Published:** 2023-02-01

**Authors:** Vassiliki Siafaka, Orestis Tsonis, Christos Christogiannis, Katerina-Maria Kontouli, Kalypso Margariti, Zoe Barbalia, Stefanos Flindris, Eleni Manifava, Kasmiria Ioanna Paschopoulou, Spyros Tzioras, Maria Baltogianni, Dimitris Mavridis, Minas Paschopoulos

**Affiliations:** School of Health Sciences, University of Ioannina, Ioannina, Greece; Assisted Conception Unit, Guy's and St Thomas’ NHS Foundation Trust, London, UK; Evidence Synthesis Methods Team, Department of Primary Education, University of Ioannina, Ioannina, Greece; Department of Obstetrics and Gynecology, University Hospital of Ioannina, Ioannina, Greece; Faculty of Medicine, School of Health Sciences, University of Ioannina, Ioannina, Greece; Private Sector, Ioannina, Greece; Neonatal Intensive Care Unit, University Hospital of Ioannina, Ioannina, Greece; Department of Obstetrics and Gynecology, Faculty of Medicine, School of Health Sciences, University of Ioannina, Ioannina, Greece

**Keywords:** COVID-19 pandemic, Pandemic-Related Pregnancy Stress Scale, Pandemic-Related Postpartum Stress Scale, psychometric properties, second wave

## Abstract

**Background:**

The COVID-19 pandemic has affected perinatal mental health. Reliable tools are needed to assess perinatal stress during pandemic situations.

**Aims:**

To assess the psychometric properties of the Greek versions of the Pandemic-Related Pregnancy Stress Scale (PREPS) and the Pandemic-Related Postpartum Stress Scale (PREPS-PP) and to explore the associations between women's characteristics and perinatal stress during the second pandemic wave.

**Methods:**

The PREPS and PREPS-PP were completed by 264 pregnant and 188 postpartum women, respectively, who also completed the State-Trait Anxiety Inventory (STAI) and the Edinburgh Perinatal Depression Scale (EPDS).

**Results:**

The internal consistency was similar for PREPS and PREPS-PP. It was good for preparedness stress (a = 0.77 and α = 0.71, respectively) and infection stress (α = 0.83 for both scales) but low for positive appraisal (α = 0.46 and α = 0.41, respectively). Of the pregnant women, 55.33% and 55.27%, respectively, reported scores of ≥40 on STAI-S and STAI-T, and the respective percentages for the postpartum women were 47.34% and 46.80%. In addition, 14.39% of the pregnant women and 20.74% of the postpartum women scored ≥13 on the EPDS. Higher preparedness stress on PREPS and PREPS-PP was associated with primiparity (*P* = 0.022 and *P* = 0.021, respectively) and disrupted perinatal care (*P* = 0.069 and *P* = 0.007, respectively). In postpartum women, higher infection stress was associated with chronic disease (*P* = 0.037), primiparity (*P* = 0.02) and perceived risk of infection (*P* = 0.065). Higher score on infection stress was associated with disrupted perinatal care in both groups (*P* = 0.107 and *P* = 0.010, respectively).

**Conclusions:**

The Greek versions of PREPS and PREPS-PP are valid tools for the assessment of women at risk of perinatal stress during a health crisis.

Since the beginning of 2020, the COVID-19 pandemic has spread rapidly around the world, causing a global health crisis with detrimental effects on society and the economy.^1^ The initial phase was followed by a variety of rapid changes in people's daily lives and an increase in their knowledge about the virus, but there were serious levels of misinformation, resulting in raised concerns and uncertainty.^2^

According to the World Health Organization, based on the results of 577 systematic reviews, with or without meta-analysis, the COVID-19 pandemic has been associated with an increase in the incidence of mental health problems, mainly anxiety and depression, especially in certain population groups, such as people with pre-existing mental health disorders.^3^

Pregnant and postpartum women are a particularly vulnerable group because of the physical and psychological changes associated with pregnancy, and the need to adjust to the constraints set by the pandemic is likely to aggravate their vulnerability.^4,5^ The stressors that affect mental health in this extremely delicate period in a woman's life include psychological factors (e.g. concern for both their own physical health and that of the fetus and/or neonate, insecurity regarding the maternal role, and changes in lifestyle, self-image and identity),^5^ obstetric factors (e.g. previous miscarriages, *in vitro* fertilisation procedures (IVF), pregnancy complications)^6^ and possible additional stressful events^7^ (e.g. serious physical illness, loss of a loved one).

Pregnancy-specific stress is related to concerns of women about their physical changes, the birth process, the health and well-being of the baby, breast-feeding experiences, raising children, and possible changes in interpersonal relationships.^8^ It has been well documented that this type of stress is a strong predictor of preterm labour, a major cause of neonatal morbidity and mortality.^9^ Moreover, it is recognised that the psychological well-being of women during the antenatal and postnatal period is crucial, as the development of mental disorders in this period may have a significant impact on the quality of mother–infant bonding and on the subsequent physical, cognitive and emotional development of the child.^5,10^

Beyond anticipated pregnancy worries, the pandemic has exacerbated women's perinatal stress by posing additional major stressors. Pregnant and postpartum women are likely to experience fear of COVID-19 infection, both of themselves and of the fetus and/or newborn infant, and concerns about the possible effects of the infection on both.^11^ The public health protection measures imposed and the worsening of the pandemic constituted additional stressors, as they included mandatory social isolation, transportation restrictions, curfews and alternative working arrangements, giving rise to possible income loss, disruption to the daily routine and increased childcare responsibilities, and the pandemic may have resulted in the actual or threatened loss of loved ones.^12^ In addition, pandemic-related changes in medical care provision, including the introduction of telemedicine, reduced access to antenatal and postnatal care, restricted the number of visitors permitted during hospital admission, and shortened the duration of hospital stay, all of which affected both the quality of life and the mental health of women during pregnancy and childbirth.^13^

Studies conducted during the first months of the pandemic in various countries demonstrated higher levels of stress and reduced psychological well-being among pregnant women and new mothers,^5,12,14^ compared with similar groups that had been assessed before the pandemic.^15^ A meta-analysis of 23 studies involving a total of 20 567 pregnant and postpartum women showed that the prevalence of anxiety and depression in pregnant women was 37% and 31%, respectively, whereas the prevalence of depression in postpartum women was 22%.^16^ Similarly, a recent scoping review concluded that pregnant women experienced high levels of anxiety and depression during the COVID-19 pandemic.^17^

As pointed out in a comprehensive scoping review of studies about the direct and indirect impact of the COVID-19 pandemic on perinatal health, it is important in the interpretation of these studies to take into consideration the respective epidemiological data of each country over time and a variety of other country-specific factors.^11^ There is a research gap in our understanding of the long-term impact of the pandemic, and further studies are needed in the face of a crisis that is probably ongoing.^18^ Comparison of the cumulative long-term impact of the COVID-19 pandemic on the perinatal mental health in women from various different countries is considered a primary goal.^19^

## The Greek situation

At the beginning of the pandemic, Greece imposed the necessary restrictions in a timely manner, and the compliance of the citizens was exemplary. Regarding the psychological impact of COVID-19 on Greek pregnant and postpartum women, high levels of anxiety were observed in studies conducted during the initial phase of the pandemic (March to September 2020),^20,21^ when the total confirmed number of cases of the disease in Greece was approximately 3400, with 190 deaths, and morbidity and mortality rates were 0.32 per 1000 individuals and 0.02 per 1000 individuals, respectively.^22^

The limited data available on perinatal stress, anxiety and depression in Greek women during the second year of the pandemic document a high prevalence of severe anxiety and depression,^23^ but without significant differences compared with the first wave of the pandemic, and a high prevalence of perinatal stress in pregnant women.^24^ Evidence from a study of postpartum women revealed increased pandemic-related stress associated with economic changes and fear of infection.^25^ During that time, a harsh lockdown was imposed in Greece, lasting from 6 November 2020 to 13 May 2021, which included strict restrictive measures, at a time when the fatigue and psychological effects of the first lockdown were already evident.

The present study was carried out from January to the end of May 2021, when the escalation of COVID-19 was high; there were about 403 000 confirmed cases of the disease in Greece and about 12 000 deaths, and the morbidity and mortality rates were 38 per 1000 individuals and 1 per 1000 individuals, respectively.^22^ The COVID-19 vaccination programme started in January 2021, but, by the end of May 2021, vaccination rates were still very low. Moreover, vaccination had not yet been approved for women during pregnancy and lactation, and there was uncertainty about the effects of SARS-CoV-2 infection on pregnant women and the fetus.

The aims of this study conducted during that period were: (a) to assess the psychometric characteristics of the Greek version of the Pandemic-Related Pregnancy Stress Scale (PREPS) developed by Preis and colleagues in 2020,^26^ and the Pandemic-Related Postpartum Stress Scale (PREPS-PP) developed by Levinson and colleagues in 2021;^27^ (b) to assess the levels of pandemic-related perinatal stress experienced by pregnant women and by women in the first month after childbirth by the administration of the PREPS and PREPS-PP, respectively; and (c) to explore the associations of demographic and obstetric characteristics with anxiety, perinatal depression and pandemic-related perinatal stress.

## Method

### Participants

This cross-sectional study was conducted in-person with 264 pregnant women who were attending an obstetrics and gynaecology clinic for regular perinatal examination and 188 women in the first month postpartum. Exclusion criteria were: age <18 years, gestational age <12 weeks in pregnant women, confirmed infection with SARS-CoV-2 during pregnancy, a history of psychiatric disorder, and incapacity to read and write Greek for any reason. All the participants provided written informed consent. The authors assert that all procedures contributing to this work comply with the ethical standards of the relevant national and institutional committees on human experimentation and with the Helsinki Declaration of 1975, as revised in 2008. All procedures involving human subjects/patients were approved by the Ethics Committee of the University Hospital of Ioannina (reference number: 2/18-1-2021, no11).

### Tools

Sociodemographic and obstetric factors were recorded: maternal age, family status, educational level, occupation, history of chronic disease, parity (primiparity or multiparity), history of miscarriages, gestational age, IVF, complications during pregnancy, fetal pathology and changes in perinatal visits to the obstetrician. The participants were asked whether they had been close to someone with COVID-19 and questioned on their perceived risk of infection of COVID-19, and possible income loss due to the pandemic was recorded.

The following instruments were administered on paper in a face-to-face data collection process.

#### PREPS and PREPS-PP

The Greek versions of PREPS^4,26^ and the modified postpartum version, adapted from the prenatal PREPS, the PREPS-PP,^27^ were administered to the pregnant and postpartum women, respectively. These are two tools which have proved to be useful for the screening of stress levels in the prenatal and postnatal period as a consequence of the pandemic. Each scale includes 15 questions that assess three dimensions: ‘preparedness stress’ (seven items), which refers to feeling unprepared for birth and the postpartum period because of the pandemic; ‘perinatal infection stress’ (five items), which refers to concerns regarding the risk of infection; and ‘positive appraisal’ (three items), which refers to the adaptive process by which stressful events, such as the pandemic, may be evaluated as beneficial. The answers are given on a five-point Likert scale ranging from 1 = very little to 5 = very much. A cut-off score ≥4 was used for identification of women with moderate to severe levels of stress. The internal consistency of the preparedness stress and infection stress dimensions in the original (English) version of the PREPS was relatively high (Cronbach's α = 0.81 and Cronbach's α = 0.86, respectively), but the internal consistency of the positive appraisal dimension was slightly lower (Cronbach's α = 0.78) than the usual criterion. However, the inter-item correlation coefficients of all items were >0.33. In the PREPS-PP version, the internal consistency ranged from Cronbach's α = 0.78 to Cronbach's α = 087 (preparedness stress α = 0.78, infection stress α = 0.80, positive appraisal α = 0.80). The Greek versions of the scales were back-translated into English by an independent professional translator. A committee of experts in the field of perinatal psychology, obstetrics and linguistics examined the conceptual, linguistic and contextual characteristics of the scales. The comprehensibility of the scales was checked by asking ten pregnant and ten postpartum women, respectively, to review the items of the scales, which led to minor expressive modifications in the final versions.

#### The State–Trait Anxiety Inventory (STAI)

STAI, developed by Spielberger (1983),^28^ assesses two types of anxiety: state anxiety (STAI-S) (as a transitory reaction to a demanding and difficult specific situation) and trait anxiety (STAI-T) (the stable tendency to experience negative emotions across many situations). It consists of 40 questions, and the answers are recorded on a four-point Likert scale ranging from 1 = not at all to 4 = very much. The score for each subscale ranges from 20 to 80, and a cut-off score of ≥45 was used to indicate moderate to high levels of anxiety. The Greek version has been validated for use in the Greek population.^29^

#### The Edinburgh Perinatal/Postnatal Depression Scale (EPDS)

EPDS^30^ is a reliable tool for detecting women at risk for perinatal depression, but it is not a diagnostic tool. It can be administered during pregnancy and in the year following the birth of a child. It is a short ten-item scale, the responses to which are given on a four-point Likert scale (0–3) indicating the severity of the symptom, and the total score ranges from 0 to 30. A cut-off score of ≥13 is most often used to indicate a fairly high possibility of depression, warranting referral to a primary mental healthcare provider. It has been translated and validated for use in the Greek population.^31^

### Statistical analysis

We analysed the data for the two groups of women, pregnant and postpartum, separately. We used Pearson correlation to test for associations between continuous variables, polyserial correlations between a continuous and an ordinal variable, and biserial correlation between a continuous and a binary variable.

We used Cronbach's α and confirmatory factor analysis (CFA) to assess the internal consistency of the PREPS and PREPS-PP items in each of the three dimensions. We used item response theory (IRT),^32^ where the items in each domain of the PREPS and PREPS-PP instruments are indicators of that domain and to test their unidimensionality, i.e. whether they all measure the same hypothetical construct within each domain. Cronbach's α indicates how closely related are a set of items as a group; it ranges between 0 and 1, with values >0.7 suggesting an acceptable level of reliability and values >0.8 suggesting a very good fit. We applied CFA using the diagonally weighted least squares estimator. We used the chi-squared test, the comparative fit index (CFI), the Tucker–Lewis index (TLI) and the root mean square error of approximation (RMSEA) to evaluate the fit of the model.

IRT is a mathematical model that explores the associations between the probabilities of the responses of individuals and the characteristics of items and the level of the respondent on the hypothetical construct measured by each domain. IRT considers both the difficulty and the discriminating ability (the ability to differentiate between subjects of different levels on the hypothetical construct) of each item; this differentiates it from the classical methods of analysing Likert-type data, where the same weight is given to all questions and the responses are treated on an interval scale. It should be noted that CFA also treats Likert-type items on an interval scale. In practice, the values on a Likert scale are not necessarily equidistant (e.g. the distance in choice between responses 1 and 2 is not necessarily the same as that between responses 2 and 3). Discrimination parameters close to zero are indicative of an item not measuring the same hypothetical construct as the rest of the items within the dimension. IRT also calculates factor scores for each dimension, which can be used in further analysis instead of the average of the items that is typically used.

Multivariable linear regression analysis was used to explore the respective associations of STAI-S, STAI-T, EPDS, and demographic and obstetric characteristics with each PREPS and PREPS-PP dimension, using the following covariates: age, parity, obstetric risk, chronic disease, cancelled perinatal appointments, income loss, perceived risk of infection, STAI-S, STAI-T and EPDS. The factor scores of the two dimensions of PREPS and PREPS-PP (preparedness stress and infection stress) were used as outcomes variables, assuming that these were measured without error. For each analysis, all the variables were entered simultaneously into an initial model, and a backward elimination procedure was applied using the Akaike information criterion (AIC).

## Results

### Sociodemographic and clinical characteristics

The study sample consisted of 452 women, of whom 264 (58.4%) were pregnant and 188 (41.6%) were mothers in the postpartum period, with a mean age of 32.99 ± 5.24 years (range 18–44 years) and 33.67 ± 5.07 years (range 20–48 years), respectively. Table 1 shows the demographic and clinical characteristics of the study population. With regard to the group of pregnant women, 68.3% had a high level of education and 51.1% had no other children. Of this group, 23.5% had a history of miscarriage, 51.3% had complications during pregnancy and 5.97% had a history of an underlying medical condition associated with higher risk for severe COVID-19, according to the Centers for Disease Control and Prevention (e.g. chronic lung disease, obesity, diabetes mellitus, cancer, immunodeficiency disorders). The pregnant women reported a variety of stressful conditions, as 28.5% had experienced pandemic-related income loss (moderate to very much), 84.7% reported cancelled perinatal care appointments or changes due to the pandemic, and 42.5% considered that there was a significant possibility (moderate to very much) of being infected by the virus (Table 1).

**Table 1:** Sociodemographic and clinical characteristics of the study sample (*N* = 452)

	Pregnant women (*N* = 264)	Postpartum women (*N* = 188)	Overall (*N* = 452)
Age, years (mean ± s.d.)	32.99 ± 5.24	33.67 ± 5.07	32.27 ± 5.17
	Range 18–44	Range 20–48	Range 18–48
Gestational age	33.10 ± 6.37	37.72 ± 2.53	34.96 ± 5.65
≤36 weeks, *N* (%)	159 (61.6)	28 (16.2)	187 (43.4)
>36 weeks, *N* (%)	99 (38.4)	145 (83.8)	244 (56.6)
Marital status, *N* (%)			
Single	21 (8.0)	10 (5.5)	31 (7.0)
Married	243 (92.0)	173 (94.5)	410 (93.0)
Occupation, *N* (%)			
Unemployed	65 (24.9)	58 (31.0)	123 (27.5)
Private employee	110 (42.1)	69 (36.9)	179 (39.9)
In suspension (due to COVID-19)	12 (4.6)	−	12 (2.7)
Civil servant	37 (14.2)	31 (16.6)	68 (15.2)
Freelance	31 (11.9)	25 (13.4)	56 (12.5)
Farmer	6 (2.3)	4 (2.1)	10 (2.2)
Education, *N* (%)			
Primary school	3 (1.2)	3 (1.61)	6 (1.3)
High school	80 (30.5)	60 (32.3)	140 (31.3)
College/university	124 (47.3)	81 (43.5)	205 (45.8)
Masters degree/PhD	55 (21.0)	42 (22.6)	97 (21.6)
Parity, *N* (%)			
Nulliparous	134 (51.1)	88 (47.6)	222 (49.7)
Multiparous	128 (48.9)	97 (52.4)	225 (50.3)
Multiple gestation, *N* (%)			
Yes	10 (4.0)	14 (9.0)	24 (5.9)
No	240 (96.0)	141 (91.0)	381 (94.1)
IVF, *N* (%)			
Yes	17 (6.5)	22 (12.1)	39 (8.8)
No	243 (93.5)	160 (87.9)	403 (91.2)
Miscarriages, *N* (%)			
Yes	62 (23.5)	41 (21.9)	103 (22.8)
No	202 (76.5)	146 (78.1)	348 (77.2)
Income loss *N* (%)			
Not at all	110 (42.5)	55 (29.9)	165 (37.2)
Little	75 (29.0)	54 (29.3)	129 (29.1)
Enough	56 (21.6)	50 (27.2)	106 (24.0)
A lot	12 (4.6)	12 (6.5)	24 (5.4)
Very much	6 (2.3)	13 (7.1)	19 (4.3)
Complications during pregnancy, *N* (%)			
Yes	109 (51.3)	70 (37.4)	179 (39.7)
No	155 (58.7)	117 (62.6)	272 (60.3)
Chronic disease, *N* (%)			
Yes	100 (37.9)	61 (32.6)	161 (35.7)
No	164 (62.1)	126 (67.4)	290 (64.3)
Medical conditions associated with higher risk for severe COVID-19, *N* (%)			
Yes	27 (5.97)	14 (3.09)	41 (9.07)
No	237 (94.03)	174 (96.91)	411 (90.93)
Fetus pathology, *N* (%)			
Yes	3 (1.1)	4 (2.1)	7 (1.6)
No	261 (98.9)	183 (97.9)	444 (98.4)
Close person infected, *N* (%)			
Yes	7 (2.7)	4 (2.2)	11 (2.4)
No	256 (97.3)	182 (97.8)	438 (97.6)
Perceived risk of infection, *N* (%)			
Not at all	30 (11.6)	15 (8.1)	45 (10.2)
Little	119 (45.9)	65 (35.2)	184 (41.4)
Enough	94 (36.3)	90 (48.6)	184 (41.4)
A lot	10 (3.9)	11 (5.9)	21 (4.7)
Very much	6 (2.3)	4 (2.2)	10 (2.3)
Cancelled medical appointments, *N* (%)			
Yes	222 (84.7)	135 (73.0)	357 (79.9)
No	40 (15.3)	50 (27.0)	90 (20.1)

IVF, *in vitro* fertilisation.

Concerning the postpartum women, 66.1% had completed at least university studies, 47.6% had no other children, 21.9% had undergone previous miscarriages, 37.4% had complications during pregnancy and 3.09% had a history of an underlying disease associated with a high risk of developing serious illness from COVID-19. Most of these women (56.7%) reported a significant perceived risk of infection, 40.8% reported income loss caused by the pandemic outbreak and 73.0% had experienced disruptions in maternity care services due to COVID-19 (Table 1).

### Internal consistency of the PREPS and PREPS-PP

The Cronbach's α coefficients for the three dimensions of PREPS were: 0.77 for preparedness stress, 0.83 for infection stress and 0.46 for positive appraisal. The Cronbach's α was lower than the acceptable threshold for the dimension of positive appraisal (Table 2).

**Table 2:** Scores on PREPS, PREPS-PP, STAI and EPDS in the two groups of women

	Pregnant women (*N* = 264) mean ± s.d. (Cronbach's α)	Postpartum women (*N* = 188) mean ± s.d. (Cronbach's α)
PREPS/PREPS-PP (range 1–5)		
Preparedness stress	2.74 ± 0.90 (0.77)	2.41 ± 0.91 (0.71)
Infection stress	2.90 ± 1.00 (0.83)	3.01 ± 0.93 (0.83)
Positive appraisal	2.32 ± 1.01 (0.46)	2.76 ± 1.10 (0.43)
Total stress	2.71 ± 0.79 (0.88)	2.68 ± 0.75 (0.86)
STAI-S (range 20–80)	43.33 ± 9.73 (0.85)	42.08 ± 10.10 (0.88)
≥40, *N* (%)	146 (55.33)	89 (47.34)
≥45, *N* (%)	107 (40.53)	64 (34.04)
STAI-T (range 20–80)	42.06 ± 6.52 (0.76)	41.22 ± 6.90 (0.79)
≥40, *N* (%)	138 (55.27)	88 (46.80)
≥45, *N* (%)	91 (34.46)	53 (28.19)
EPDS (range 0–30)	7.68 ± 5.38 (0.86)	7.41 ± 6.05 (0.88)
≥13, *N* (%)	38 (14.39)	39 (20.74)

PREPS, Pandemic-Related Pregnancy Stress Scale; PREPS-PP, Pandemic-Related Postpartum Stress Scale; STAI-S, State-Trait Anxiety Inventory-State; STAI-T, State-Trait Anxiety Inventory-Trait; EPDS, Edinburgh Postnatal Depression Scale.

Similarly, the Cronbach's α values for the dimensions of PREPS-PP were: 0.71 for preparedness stress, 0.83 for infection stress and 0.43 for positive appraisal. Again, the Cronbach's α was lower than the acceptable threshold for the dimension of positive appraisal (Table 2).

The same results were found for both PREPS and PREPS-PP from the discrimination parameters derived from the IRT model for positive appraisal, in which two of three items could not discriminate between individuals at different levels, indicating that these items do not measure the same hypothetical construct (see Supplementary Appendix Table 1 available at https://doi.org/10.1192/bjo.2022.635). As shown in parentheses in Supplementary Table 1, the results are given assuming a unidimensional model; the two items that discriminate better in positive appraisal show a small discrimination parameter and hence are responsible for the low Cronbach's α in both PREPS and PREPS-PP, as they do not measure the same hypothetical construct as the remaining items of PREPS and PREPS-PP.

### Confirmatory factor analysis

**Fig. 1:**
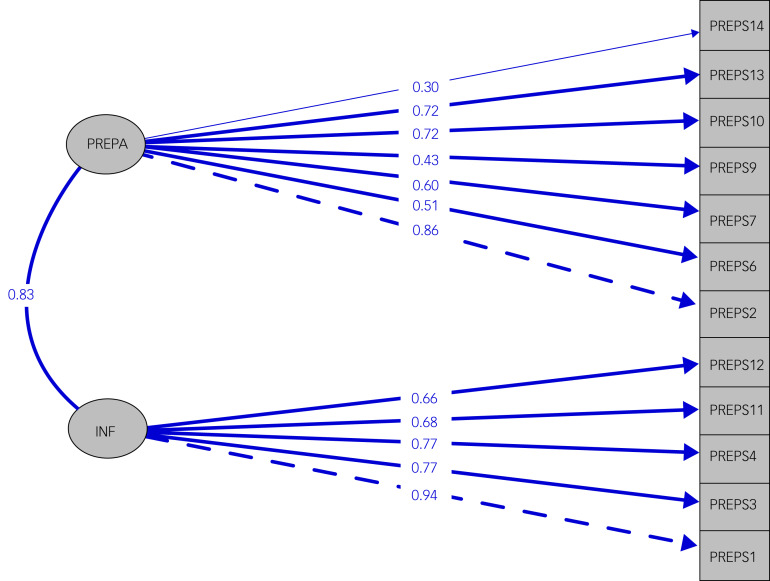
Confirmatory factor analysis of the PREPS: the two-factor model structure and item loadings of PREPS. PREPA, Preparedness Stress; INF, Infection Stress.

**Fig. 2:**
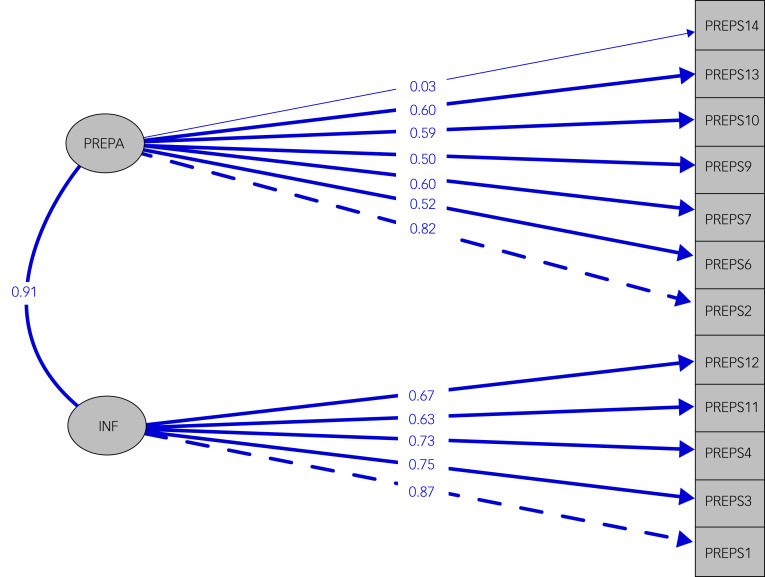
Confirmatory factor analysis of the PREPS-PP: the two-factor model structure and item loadings of PREPS-PP. PREPA, Preparedness Stress; INF, Infection Stress.

CFA was applied considering (a) a model with two factors, preparedness stress and infection stress; (b) a model with all three factors; and (c) a one-factor model, where all items are indicators of the same latent construct, for both PREPS and PREPS-PP. The three-factor model fitted less well than the two-factor and one-factor models, as demonstrated by the CFI and TLI indices, which did not reach the threshold of 0.90 and had the highest RMSEA for both prenatal and postpartum samples (Figs. 1, 2 and Table 3).

**Table 3:** Confirmatory factor analysis for PREPS and PREPS-PP in samples of pregnant and postpartum women

		χ^2^	d.f.	CFI	TLI	RMSEA
PREPS	Three-factor model	452.702	90	0.897	0.880	0.124
	Two-factor model	279.630	55	0.918	0.902	0.125
	One-factor model	376.129	91	0.919	0.907	0.110
PREPS-PP	Three-factor model	406.440	90	0.852	0.827	0.140
	Two-factor model	170.366	55	0.929	0.915	0.107
	One-factor model	347.780	91	0.880	0.862	0.125

PREPS, Pandemic-Related Pregnancy Stress Scale; PREPS-PP, Pandemic-Related Postpartum Stress Scale; CFI, comparative fit index; TLI, Tucker–Lewis index; RMSEA, root-mean-square error of approximation.

More precisely, the two-factor model was better than the one-factor model for the postpartum sample, but in the prenatal sample they had similar performance. The three-factor solution gave the worst fit in terms of Cronbach's α and IRT results. Considering all this information, we excluded ‘positive appraisal’ from further analysis for both study populations.

### Magnitude of stress

The PREPS dimension with the lowest mean score was positive appraisal (2.32 ± 1.01) and that with the highest was perinatal infection stress (2.90 ± 1.00) (Table 2). For the postpartum women, the PREPS-PP dimension with the lowest mean score was preparedness stress (2.41 ± 0.91) and that with the highest was infection stress (3.01 ± 0.93). The cut-off score of ≥4 was used for detecting moderate to severe levels of stress. Of the pregnant women, 8.07% recorded high levels of preparedness stress and 14.01% recorded high levels of perinatal infection stress, whereas the percentages in the postpartum women were 4.78% and 12.76%, respectively. The percentage of pregnant women who reported a score of ≥4 on the positive appraisal item (which reflects a more positive cognitive evaluation of having a baby during the pandemic) was 5.3%, whereas that of the postpartum women was 11.17%.

### Anxiety and depression symptoms

Table 2 shows the scores of the study population on the mental health scales. Regarding anxiety in pregnant women, the mean scores were 43.33 ± 9.73 on STAI-S and 42.06 ± 6.52 on STAI-T. Using a cut-off score of ≥40, which has been used in similar studies, 55.33% and 55.27% of the pregnant women recorded ≥40 on STAI-S and STAI-T, respectively, and 40.53% and 34.04% of pregnant women recorded ≥45 on STAI-S and STAI-T, respectively, indicating moderate to higher anxiety. With respect to antenatal depression, the total mean score on EPDS was 7.68 ± 5.38, with 14.39% reporting a mean score of ≥13 (Τable 2).

In the postpartum women, the mean score on STAI-S was 42.08 ± 10.10 and that on STAI-T was 41.22 ± 6.90. Scores of STAI-S ≥40 and STAI-T ≥40 were recorded by 47.34% and 46.80% of the postpartum women, respectively, and of STAI-S ≥45 and STAI-T ≥45 by 34.46% and 28.19%, respectively.

Concerning postnatal depression, the total mean score of postpartum women on EPDS was 7.41 ± 6.05, and 20.74% recorded a mean score of ≥13, corresponding to a high possibility of depression (Τable 2).

### Correlation coefficients

The factor scores from the IRT model were used to explore associations between the two dimensions of the PREPS and PREPS-PP and the women's characteristics for each group, pregnant and postpartum. Table 4 presents the Pearson, polyserial and polychoric correlations between the two-factor scores and various characteristics for both groups.

**Table 4:** Correlation of the factor scores on PREPS and PREPS-PP with demographic and clinical characteristics of the pregnant and postpartum women and STAI-S, STAI-T and EPDS

	PREPS			PREPS-PP		
	Preparedness stress	Infection stress	Total stress	Preparedness stress	Infection stress	Total stress
Age	−0.14	0.03	−0.06	−0.08	−0.07	−0.08
Gestational age	0.03	<0.01	<0.01	−0.12	−0.05	−0.07
Parity	0.09	−0.08	−0.01	−0.16	−0.15	−0.13
IVF	−0.02	0.02	0.02	0.08	0.11	0.10
Obstetric risk	0.04	0.01	−0.04	−0.11	−0.15	−0.15
Chronic disease	0.02	−0.06	<0.01	0.03	0.10	0.07
Miscarriages	0.01	−0.05	0.03	0.02	0.05	0.06
Close person infected	−0.04	−0.02	0.04	0.05	0.08	0.09
Cancelled perinatal care appointments	0.19	0.19	0.21	0.28	0.32	0.32
Income loss	0.31	0.20	0.25	0.14	0.22	0.21
Perceived risk of infection	0.21	0.25	0.24	0.15	0.18	0.21
STAI-S	0.34	0.25	0.32	0.37	0.32	0.36
STAI-T	0.31	0.22	0.29	0.21	0.17	0.20
EPDS	0.27	0.14	0.24	0.19	0.22	0.22

PREPS, Pandemic-Related Pregnancy Stress Scale; PREPS-PP, Pandemic-Related Postpartum Stress Scale; IVF, *in vitro* fertilisation; STAI-S, State-Trait Anxiety Inventory-State; STAI-T, State-Trait Anxiety Inventory-Trait; EPDS, Edinburgh Postnatal Depression Scale.

For the two dimensions of PREPS, the factors with correlations ≥0.2 were cancelled perinatal care appointments, income loss, perceived risk of infection, and scores on STAI-S, STAI-T and EPDS, with STAI-S having the highest correlations with preparedness stress and infection stress.

The same applied for the two dimensions of PREPS-PP, with the same variables having correlation coefficients of ≥0.2 and STAI-S having the highest.

### Linear regression analysis

To explore further the associations of the two-factor scores on the PREPS and PREPS-PP with several of the women's characteristics and indices of psychological distress, multiple regression analysis models were applied using the two-factor scores as outcomes and the women's characteristics as covariates. Tables 5 and 6 show the selected variables for each of the two-factor scores. In the pregnant women, the covariates that were common to both models were STAI-S and cancelled perinatal care appointments. Three of ten variables selected as potential covariates were common to both models for the postpartum group, specifically STAI-S, parity and cancelled perinatal care appointments.

**Table 5:** Statistically significant effects on two-factor score dimensions (PREPS) in pregnant women's (*N* = 264)

*R*^2^_adj_ = 0.21	B	s.e.	*P*-value
**Preparedness stress factor score**			
Age	−0.023	0.010	0.026
STAI-S	0.019	0.007	0.008
STAI-T	0.020	0.011	0.056
Parity	−0.259	0.112	0.022
Income loss	0.247	0.270	0.361
	0.205	0.234	0.381
	0.673	0.226	0.003
	0.448	0.172	0.010
Cancelled perinatal care appointments	0.306	0.168	0.069
**Infection stress factor score**			
STAI-S	0.019	0.006	0.002
Cancelled perinatal care appointments	0.288	0.178	0.107
**Total stress factor score**			
Age	−0.019	0.011	0.095
STAI-S	0.027	0.006	<0.001
Income loss	0.105	0.294	0.721
	0.012	0.253	0.961
	0.490	0.242	0.045
	0.245	0.185	0.187
Cancelled perinatal care appointments	0.281	0.178	0.116

*R*^2^_adj_, adjusted *R*^2^; PREPS, Pandemic-Related Pregnancy Stress Scale; STAI-S, State-Trait Anxiety Inventory-State; STAI-T, State-Trait Anxiety Inventory-Trait; B, unstandardised coefficient for the PREPS factor scores outcomes after backward elimination with AIC as stopping rule criterion. Note that the *P*-value considered from the AIC stopping rule is lower than approximately 0.157. This explains why some variables stayed in the model although they had a *P*-value larger than 0.05.

**Table 6:** Statistically significant effects on two-factor score dimension in PREPS-PP in postpartum women (*N* = 188)

*R*^2^_adj_ = 0.21	B	s.e.	*P*-value
**Preparedness stress factor score**			
STAI-S	0.029	0.006	<0.001
Parity	−0.297	0.127	0.021
Cancelled perinatal care appointments	0.387	0.141	0.007
**Infection stress factor score**			
STAI-S	0.021	0.007	0.003
Parity	−0.332	0.141	0.020
Perceived risk of infection	0.761	0.409	0.065
	0.457	0.339	0.180
	0.071	0.268	0.791
	0.237	0.171	0.169
Cancelled perinatal care appointments	0.403	0.155	0.010
Chronic disease	0.298	0.142	0.037
**Total stress factor score**			
STAI-S	0.028	0.007	<0.001
Parity	−0.328	0.140	0.021
Perceived risk of Infection	0.770	0.407	0.061
	0.292	0.337	0.387
	0.113	0.266	0.672
	0.231	0.170	0.177
Cancelled perinatal care appointments	0.429	0.154	0.006
Chronic disease	0.265	0.141	0.062

*R*^2^_adj_, adjusted *R*^2^; PREPS-PP, Pandemic-Related Postpartum Stress Scale; STAI-S, State-Trait Anxiety Inventory-State; STAI-T, State-Trait Anxiety Inventory-Trait; B, unstandardised coefficient for the PREPS factor scores outcomes after backward elimination with AIC as stopping rule criterion. Note that the *P*-value considered from the AIC stopping rule is lower than approximately 0.157. This explains why some variables stayed in the model although they had a *P*-value larger than 0.05.

In the pregnant women, higher preparedness stress was reported by primiparous women (B = −0.259, *P* = 0.022), women with loss to their income (B = 0.247, *P* = 0.361) and those who reported cancelled perinatal care appointments (B = 0.306, *P* = 0.069). In addition, higher perinatal infection stress was recorded by pregnant women who had reported cancelled perinatal care appointments (B = 0.288, *P* = 0.107) (Table 5).

In the postpartum women, higher preparedness stress was reported by primiparous women (B = −0.297, *P* = 0.021) and those who had perinatal care appointments cancelled (B = 0.387, *P* = 0.007). Higher infection stress was recorded by those with history of chronic disease (B = 0.298, *P* = 0.037), those who were primiparous (B = −0.332, *P* = 0.020), those with at least some perceived risk of infection (B = 0.761, *P* = 0.065) and those who reported cancelled perinatal care appointments (B = 0.403, *P* = 0.010) (Table 6).

## Discussion

The aims of this study were to assess the psychometric properties of the Greek versions of the PREPS and PREPS-PP, to estimate the levels of pandemic-related perinatal stress experienced by pregnant and postpartum women during the second year of the pandemic, and to explore the associations of demographic and obstetric characteristics with anxiety, perinatal depression and pandemic-related perinatal stress.

The results showed that the internal consistency was high for the preparedness stress and perinatal infection stress dimensions of the PREPS, but the Cronbach's α was lower than the acceptable threshold for the positive appraisal dimension. This pattern was confirmed by IRT and polychoric correlations. It therefore appears that two of the three items in the positive appraisal dimension do not measure the same hypothetical construct. These findings are consistent with the results of a similar study conducted in a Spanish sample.^33^ The validation study of the original version of PREPS^26^ in American women and those conducted for Polish^34^ and Hebrew^35^ language versions also showed that the internal consistency of the positive appraisal dimension was slightly lower than the usually accepted Cronbach's α = 0.70 criterion. Conversely, the positive appraisal dimension showed satisfactory internal validity in validation studies conducted in German^19^ and Italian population samples.^36^ According to the authors^26^ of the original version, a low value of Cronbach's α could be due to the small number of items in this dimension (three items). This is a known limitation of Cronbach's α, and Pallant and colleagues^37^ suggest not using it for scales with fewer than six items. We cannot exclude the possibility that the low internal consistency is a genuine result, and that differences in the Cronbach's α across different studies could be attributed to differences in population characteristics^38^ (e.g., cultural differences), translation of the instrument, the time and/or setting in which the instrument was delivered or other variables.

Regarding CFA, the two-factor solution gave the best results, and this was in line with the results of the Polish validation study,^24^ although small differences were found in terms of goodness-of-fit between the two- and the three-factor models. Positive appraisal was also weakly correlated with the two stress factors, preparedness stress and infection stress; for this reason, it can be treated as a separate construct.

Similar results were observed for the PREPS-PP. To the best of the authors’ knowledge, the postpartum scale has not yet been validated in other languages. The original version of the scale showed good psychometric properties in all three dimensions, and CFA confirmed a good fit on the three-factor structure.^27^ The findings from the various types of analysis that we applied (Cronbach, CFA, IRT, inter-item correlations) again showed that the Greek sample did not identify the positive appraisal dimension of the scale.

With respect to pandemic-related pregnancy stress perceptions, the dimension of PREPS with the highest mean score was perinatal infection stress. Using the cut-off score of ≥4 for detecting moderate to severe levels of stress, during the second year of the pandemic 8.07% of the pregnant women recorded a high level of preparedness stress and 14.01% a high level of perinatal infection stress. These percentages are significantly lower than those reported in a study using the same questionnaire with pregnant women conducted during the initial phase of the pandemic in the USA, in which approximately 30% and 29.1% of pregnant women reported high levels of preparedness stress and perinatal infection stress, respectively.^4^ During the same period, in a similar study conducted in Italy, high preparedness stress and infection stress were reported by 9.1% and 9.9% of pregnant women, respectively,^36^ and in May and June, 16% of a German-speaking sample reported high levels of preparedness stress and 12% reported high levels of infection stress.^19^ A recent study of 8148 pregnant women from seven high-income Western countries, using a well-fitting common path model, showed that although pregnant women experienced different levels of stress during the initial phase of the COVID-19 pandemic, stress was a strong and common predictor of anxiety and depression symptoms above the clinically defined thresholds for poor mental health.^39^

These differences in stress levels may be accounted for by the varying rigor of the measures imposed in each country and, in particular, the measures concerning antenatal and postnatal care, the capacity of the healthcare systems to meet pregnant women's needs, and possibly the style and quality of information provided by the government in each country.^40^ The low level of tolerance for uncertainty at the beginning of the pandemic and the habituation to pandemic stress over time may also explain the differences.^41^

Concerning pandemic-related postpartum stress, the dimension of PREPS-PP with the highest mean score was infection stress, with 4.78% and 12.76% of postpartum women reporting severe preparedness stress and infection stress, respectively. Research data^27^ indicate that American postpartum women reported that they experienced ‘little’ to ‘some’ pandemic-related postpartum stress, on average, which was reduced at 3 month follow-up. The findings of the present study are in line with those of a similar study^25^ conducted in postpartum women during the second wave of the pandemic using a different stress scale. One study^12^ conducted earlier in the course of the pandemic indicated that major contributors to perceived perinatal stress included financial and familial and/or social factors related to the COVID-19 restrictions.

With regard to emotional reactions to stress, and using the cut-off score of ≥40 on STAI-S and STAI-T (mild anxiety) which has been used in similar studies,^21,42^ in our study 55.33% and 55.27% of the pregnant women recorded STAI-S and STAI-T ≥40, respectively, and 40.53% and 34.46% recorded STAI-S and STAI-T ≥45 (moderate to high anxiety), respectively. In addition, 14.39% of the pregnant women indicated a fairly high possibility of perinatal depression. A significant number of the pregnant women reported persistent, intense worries related to the specific condition they were experiencing, and also a constant stable tendency to experience negative emotions across many situations. These findings indicate that during the second year of the pandemic, pregnant women in Greece demonstrated a higher prevalence of anxiety compared with the women in a previous study^21^ conducted during the first lockdown; this previous study reported that the pandemic increased anxiety rates in pregnant women in Greece but not rates of depression, which remained at the same level as those recorded before the pandemic.^43^ It has been well established that anxiety is more prevalent than depression during pregnancy in populations in Europe, with rates documented at 18% and 15%, respectively.^44^

Concerning the postpartum women in the present study, the percentages who reported moderate to high levels of anxiety were 34.04% (STAI-S) and 28.19% (STAI-T), and 20.74% screened positive for postnatal depression. This indicates that during the second wave of the pandemic, the prevalence of anxiety and depression in postpartum women in Greece was higher than that reported both before the pandemic^45^ and during the first lockdown.^20^ These findings can be explained by the fact that during this second period, women who gave birth may have been forced to recover alone or to leave the hospital early. It was possible that some had been isolated for a long time from their family, elderly parents and friends while taking care of the newborn baby. A meta-analysis of 34 studies from many countries indicated that anxiety rates were higher in studies conducted later in the pandemic, and significant differences were observed between different geographical areas, with lower rates detected in East Asia than in European countries. The variation observed in the prevalence of anxiety and depression can be explained in part by the different rates of infection and deaths from COVID-19 during the given period in each of the countries studied, the restrictive measures imposed, access to care, income, and the relevant information provided.^40^

Based on the results of multivariable regression analysis in our study population, primiparity, loss of income, cancelled perinatal care appointments and psychological distress can be considered as factors associated with preparedness stress in pregnant women, and disruption in normal perinatal care appointments and psychological distress were associated with infection stress in this group. Among the postpartum women, higher levels of preparedness stress were reported by primiparous women, those who experienced changes in maternity care conditions and those who reported psychological distress, whereas higher perinatal infection stress was associated with history of chronic disease, primiparity, cancelled perinatal care appointments and psychological distress. These findings are in agreement with results from similar studies, in which higher levels of pandemic-related perinatal stress were reported by primiparous women.^27^ The transition to motherhood is a period of crisis that is associated with a variety of changes and the need for reorganisation and adjustment to the parenting role.^46^ This process may be accompanied by parenting stress resulting from the gap between the requirements associated with the parenting role and the perceived resources for dealing with these requirements. Parenting stress may be aggravated by pandemic-related stressors,^47^ including resource constraints, rapid changes in social and economic circumstances and healthcare provision practices, and uncertainty about the future.

The present study demonstrated that women were worried about being unprepared for the birth and the care of a newborn because of the pandemic and its disruptive impact on perinatal care, a finding which is in accordance with those of previous studies.^34,40^ It is apparent that cancellation of scheduled perinatal appointments and child-related worries (e.g. inadequate childcare support) are risk factors for post-traumatic stress, anxiety and depression^13^ and are perceived by women as ‘withdrawal of care’.^48^ Women may feel less well informed and less prepared for childbirth as a consequence of the reduction of scheduled in-person visits to the doctor and, in some cases, they may feel ‘less important’ and frustrated.^26^ In addition, owing to the decreased prenatal care visits, they are likely to have concerns about the reduced opportunity to diagnose possible pregnancy complications, about the limited access to standard services, and the failure to meet their medical and support needs, all of which may lead to increased anxiety and emotional disturbance.^40^ Standard perinatal care was reformed owing to the need for prolonged physical distancing measures, and health systems were not always capable of providing routine support services to pregnant and postpartum women.^49^

The self-perceived risk of COVID-19 infection was associated with high scores on preparedness stress and infection stress among the postpartum women. As reported in a systematic review, women during pregnancy and breastfeeding expressed increased stress about becoming infected with COVID-19, and their concerns were related mainly to the welfare and health of their children.^18^ The Greek government, from the beginning of the pandemic, informed the population about the spread of COVID-19 through live broadcast daily briefings. During the second year, the pandemic spread rapidly, and emergency alerts and media campaigns were added, which probably resulted in an increase in fear and anxiety in these women. The news channels gave special coverage to cases of COVID-19 during pregnancy. The risk of infection, the daily news about the impact of the pandemic and the strict measures imposed for the protection of public health inarguably affected the mental health of the Greek people.^50^

Income loss due to the pandemic restrictions was found to be an independent predictor of pandemic-related pregnancy stress, which is consistent with other published evidence documenting the impact of financial distress due to COVID-19 on mental health of pregnant women.^51^

Not surprisingly, the results of the present study showed that the presence of chronic disease was a risk factor for the development of pandemic-related stress in postpartum women during the second year of the pandemic, as has been reported by other studies.^14^

The results of the present study highlight the magnitude of the burden of the pandemic on the mental health of Greek women during the perinatal period in the course of the second wave. Concerns related to COVID-19 itself, financial insecurity, feeling unprepared for the birth and the postpartum period because of restricted access to healthcare services, especially by primiparous women, are factors that affected perinatal mental health. Validated and reliable instruments such as PREPS and PREPS-PP can be valuable tools for health professionals to assess the perinatal stress that pregnant and postpartum women face during the pandemic. The health system should be prepared to respond comprehensively to the increased needs of this vulnerable group during the ongoing health crisis, or in the case of future massive stressful events, in order to preserve the mental health of mothers and babies. Specifically, regular mental health screening is necessary for the monitoring of symptoms of anxiety and depression, in order to provide timely intervention. The multi-level organisation of services is necessary; this should include enhancement of capabilities for internet-based screening and psychological intervention, mobile teams for prevention and intervention, and improvement of crisis response services. In addition, it is crucial to educate women on health protection measures, but also on ways of promoting their mental health. Finally, relevant educational programmes should include all those involved in the provision of perinatal services and the care of women before and after childbirth, such as obstetricians, midwives, mental health professionals, policy makers and close relatives.

### Strengths and limitations

The present study had several strengths. A key strength was the fact that we conducted three different validity analyses to explore the dimensionality of PREPS and PREPS-PP, including IRT, which not only does not give the same weight to all items but also treats the responses to items as ordinal variables and not on an interval scale. Additional strengths were the face-to-face administration of the questionnaires during routine medical visits and the recording of obstetric characteristics by an obstetrician. It is of note that most similar studies were conducted online and included only women with access to the internet and social media, and self-reported obstetric characteristics may be biased. A final strength of the study is considered to be the fact that it was conducted during the second year of the pandemic, at which time rates of confirmed COVID-19 cases and deaths were high in Greece, and the accumulated mental burden and fatigue of the population was evident.

Some limitations should be noted, however. First, the study design was cross-sectional and, therefore, determination of causality is not possible. Second, the sample was recruited from a single tertiary hospital, although this is a university hospital that covers the needs of all of north-western Greece. Third, the study design did not include a control group of age-matched women from the general population. Finally, in the regression analyses, we used the factor scores as those derived from IRT analyses. These scores are estimated from a statistical model (IRT) and, therefore, are not free of measurement error. The results were similar even when using factor scores estimated using CFA.

### Implications

The results of the present study indicate that the Greek versions of PREPS and PREPS-PP are valid, useful tools for assessment of women at risk of perinatal stress during a health crisis. The study contributes to our understanding of the psychological distress symptoms experienced by pregnant and postpartum women in Greece during the second year of the pandemic under circumstances of continued restrictive public health protection measures. Inconsistent perinatal care, perceived risk of infection, income loss, primiparity and a history of chronic disease were factors demonstrated to be associated with pandemic-related perinatal stress in these women. The implementation of special prevention programmes and the monitoring and management of perinatal mental health during the ongoing health crisis or other future massive stressful events are of paramount importance, in order to preserve the mental health of mothers and babies.

## Data Availability

The data that support the findings of this study are available from the corresponding author, V.S., upon reasonable request.

## Supplementary material

For supplementary material accompanying this paper visit http://doi.org/10.1192/bjo.2022.635. click here to view supplementary material
