# Supplementary material for: Psychometric properties of the Greek versions of the Pandemic-Related Pregnancy Stress Scale and the Pandemic-Related Postpartum Stress Scale and associated risk factors during the second year of the COVID-19 pandemic
Source: BJPsych Open. 2023 Feb 1;9(2):e25. doi: 10.1192/bjo.2022.635 (PMC9970181; doi:10.1192/bjo.2022.635)
Supplement: Supplementary file 1 [file S2056472422006354sup001.docx]

**Table S1.** IRT discrimination parameters for the three dimensions of the 15-Item PREPS and PREPS-PP for prenatal and postpartum woman, respectively. In parentheses is the discrimination parameter considered as total stress. (Appendix)

|  | **Infection Stress** | | **Positive Appraisal** | | **Preparedness Stress** | |
| --- | --- | --- | --- | --- | --- | --- |
|  | **Question** | **Discrimination** | **Question** | **Discrimination** | **Question** | **Discrimination** |
| Pregnant | Item 1 | 1.505 (1.534)  2.833 (2.443) | Item 5 | 3.784 (0.902)  3.531 (0.609) | Item 2 | 1.189 (1.580)  1.707 (2.229) |
| Postpartum |  |  |  |  |  |  |
| Pregnant | Item 3 | 3.991 (2.332)  3.861 (2.831) | Item 8 | 1.122 (0.498)  1.232 (0.248) | Item 6 | 1.360 (1.251)  1.866 (1.240) |
| Postpartum |  |  |  |  |  |  |
| Pregnant | Item 4 | 3.660 (2.510)  2.642 (2.329) | Item 15 | 0.415 (1.261)  0.235 (1.363) | Item 7 | 1.599 (1.484)  2.622 (1.607) |
| Postpartum |  |  |  |  |  |  |
| Pregnant | Item 11 | 1.391 (2.234)  1.292 (1.603) |  |  | Item 9 | 0.921 (0.885)  1.271 (1.114) |
| Postpartum |  |  |  |  |  |  |
| Pregnant | Item 12 | 1.246 (1.834)  1.385 (1.737) |  |  | Item 10 | 2.412 (2.468)  0.983 (1.491) |
| Postpartum |  |  |  |  |  |  |
| Pregnant |  |  |  |  | Item 13 | 2.765 (2.313)  1.076 (1.386) |
| Postpartum |  |  |  |  |  |  |
| Pregnant |  |  |  |  | Item 14 | 0.649 (0.674)  0.159 (0.070) |
| Postpartum |  |  |  |  |  |  |

*Abbreviation: PREPS: Pandemic-Related Pregnancy Stress; PREPS-PP: Pandemic-Related Postpartum Stress Scale*
